# Cerebral fat embolization with paroxysmal sympathetic hyperactivity syndrome and septic shock at high altitude: a case report and literature review

**DOI:** 10.1186/s41016-021-00232-6

**Published:** 2021-02-18

**Authors:** Min Li, Gang Zhu, Hao Guo, Shun Nan Ge, Guo Dong Gao, Yan Qu

**Affiliations:** Department of Neurosurgery, The Second Affiliated Hospital, Air Force Medical University, No. 569 Xinsi Road, Xi’an, 710038 China

**Keywords:** Cerebral fat embolization, High altitude, Paroxysmal sympathetic hyperactivity, Septic shock, Trauma

## Abstract

**Background:**

Cerebral fat embolism (CFE) syndrome at high altitude was rare complicated with paroxysmal sympathetic hyperactivity (PSH) syndrome and septic shock. It is a challenge to differential diagnosis and treatment at high altitude.

**Case presentation:**

This case presents a CFE with PSH and septic shock of a 23-year-old man occurred at high altitude of 3800 m above sea level, transferred by airplane successfully and cured in the department of neurosurgery, Xi’an Tangdu Hospital.

**Conclusions:**

It is key that CFE with PSH can be rapid diagnosed and treatment bundles of septic shock should be initiated as soon as possible. Early neurological rehabilitation played an important role for good outcome.

## Background

Cerebral fat embolism (CFE) usually occurs after trauma or during surgical procedures and it occurs in 0.5–3.5% of cases [1, 2]. A limited case CFE with paroxysmal sympathetic hyperactivity (PSH) has been reported [3]. We presented a complicated young case of CFE complicated with PSH and septic shock at high altitude.

## Case presentation

**Fig. 1:**
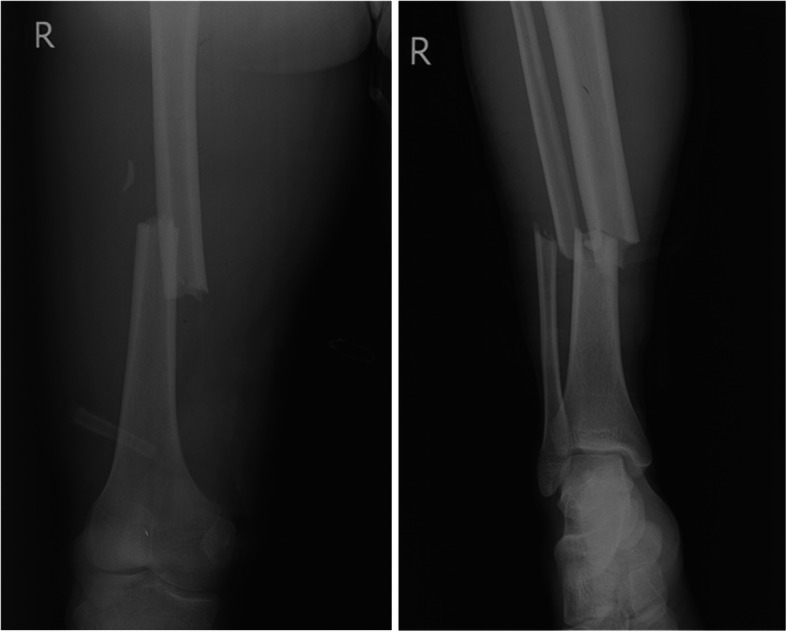
X-ray examination images of the right femoral shaft, as well as tibia and fibula obtained 2 h after the accident

**Fig. 2:**
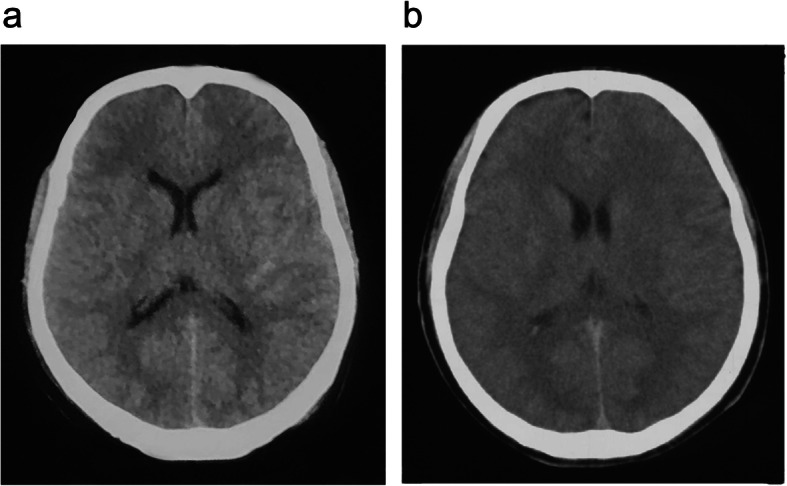
**A** Head CT obtained 11 h after the accident; the patient was agitated and lost consciousness with convulsions. **B** The head CT scan obtained 3 days after the accident, which indicated brain swelling

**Fig. 3:**
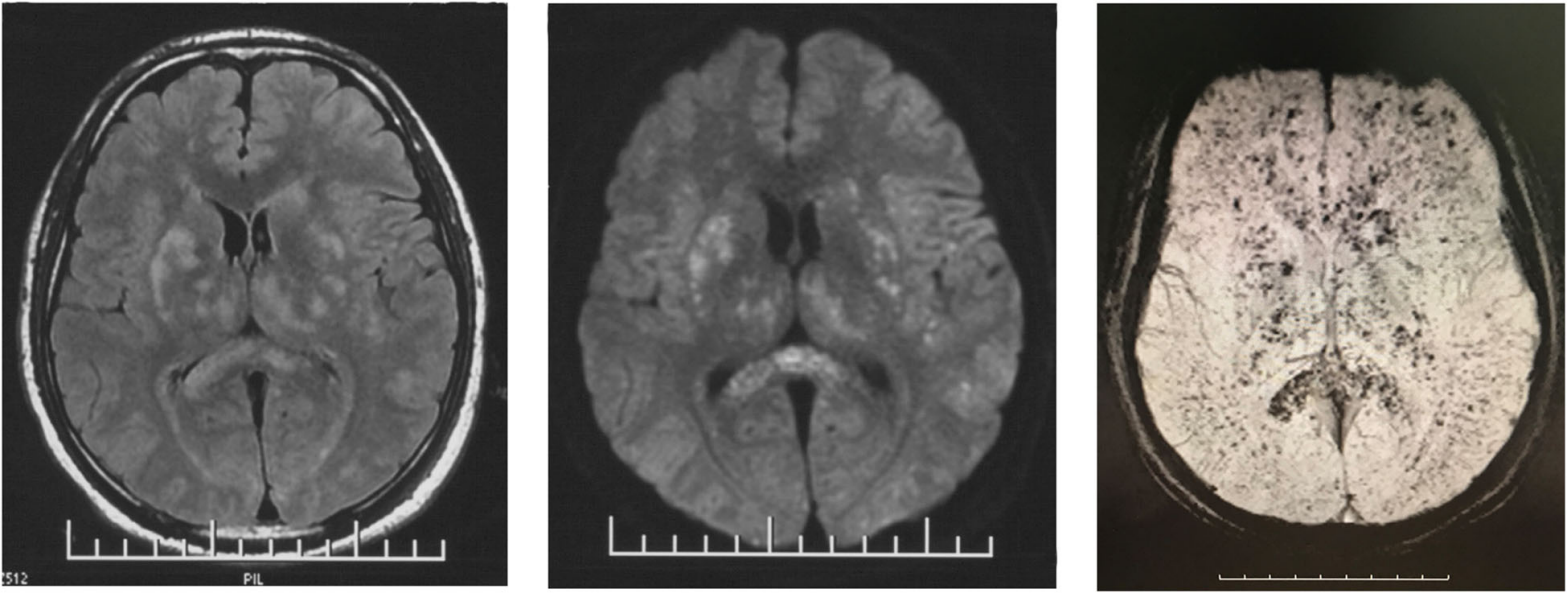
MRI scans obtained 3 days after the accident (FLAIR, DWI, SWI sequence in order)

A 23-year-old man had a right closed femoral midshaft femur, tibia, and fibula fracture (Fig. 1) from a motor vehicle accident on a height of 3800 m above sea level. He was rescued 1 h later and conscious (GCS 15). Four hours later, he was transferred to local hospital and place a proximal tibial traction pin. Eleven hours after the accident, the patient was agitated and lost consciousness with convulsions. GCS was reduced to 6. The head CT scan showed normal (Fig. 2A). Three days later, he had a fever over 39 °C and the convulsions were more frequently than before. The convulsion was controlled by propofol and sodium valproate, but consciousness was not improved (GCS 6) after suspending sedatives. Then, we did the neuroimaging examination for consciousness disorder. The head CT scan indicated brain swelling (Fig. 2B). MRI showed innumerable foci of hyperintense lesions in a “starfield” pattern on T2, FLAIR and DWI sequence images, located at the periventricular, subcortical, basal ganglia, cerebellum, and deep white matter predominantly (Fig. 3). The diagnosis maybe difficult to differentiate diffused axion injury (DAI) and CFE if only based on neuroimaging evidence. Finally, CFE was diagnosed based on clinical features and neuroimaging. The reason was illuminated in the discussion section.

**Fig. 4:**
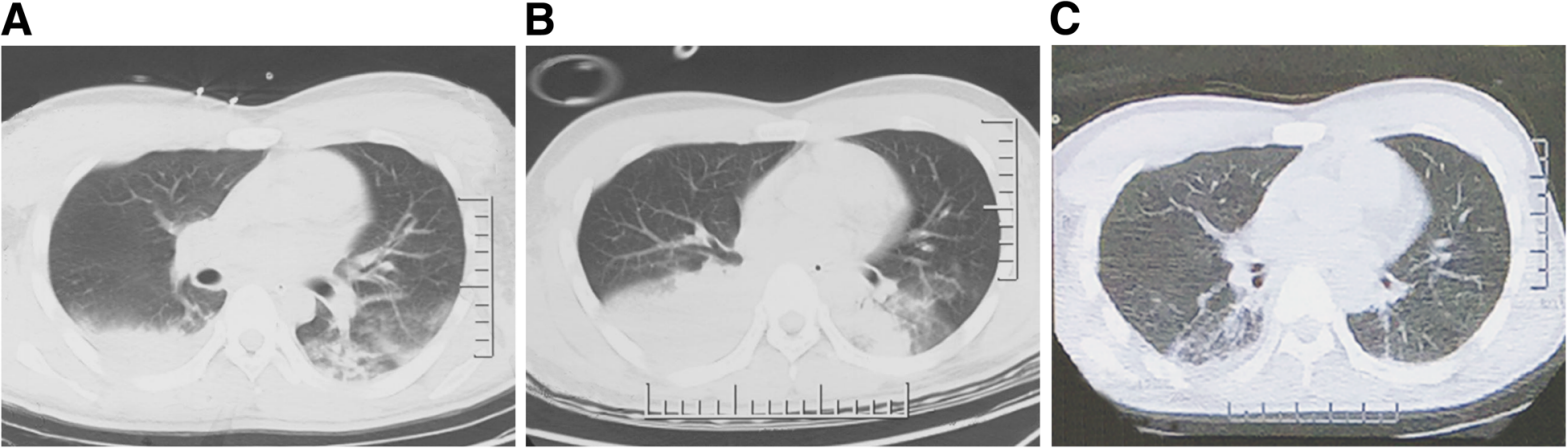
**A** The chest CT scan obtained 3 days post trauma. **B** The chest CT scan obtained 8 days post trauma. **C** The chest CT scan obtained 22 days post trauma (14 days post anti-sepsis bundles)

His core temperature was over 39 °C after 3 days post trauma. The chest CT scan indicated pneumonia (Fig. 4A) and antibiotics was administrated. More seriously, hypotension was presented (85/50 mmHg) and respirate rate was 30/min, indicating that the patient got to be septic shock caused by pneumonia. The Sequential Organ Failure Assessment (SOFA) score was 12. As to be more critical than before, he was transferred to Tang Du Hospital by air.

When arrived, he was comatose and convulsive with ictus from 8 to 10 times daily. The manifestation was the intermittent hypertension (blood pressure up to 175/90 mmHg), tachycardia (pulse up to 155/min), febrile (up to 39 °C), tachypnea (respire rate up to 45/min), and diaphoresis. Flexor posturing was existed at the same time. The duration of episodes was within 5 min. Moreover, continuous electroencephalogram (cEEG) suggested a moderate inhibition of cortex function without epileptiform discharges. PSH was diagnosed based on clinical features, imaging, and cEEG. The treatment included propranolol and midazolam. To better control the episodes, fentanyl, dexmedetomidine, and bromocriptine were administered. The episode of PSH was gradually improved, no more than 2 times per day.

**Fig. 5:**
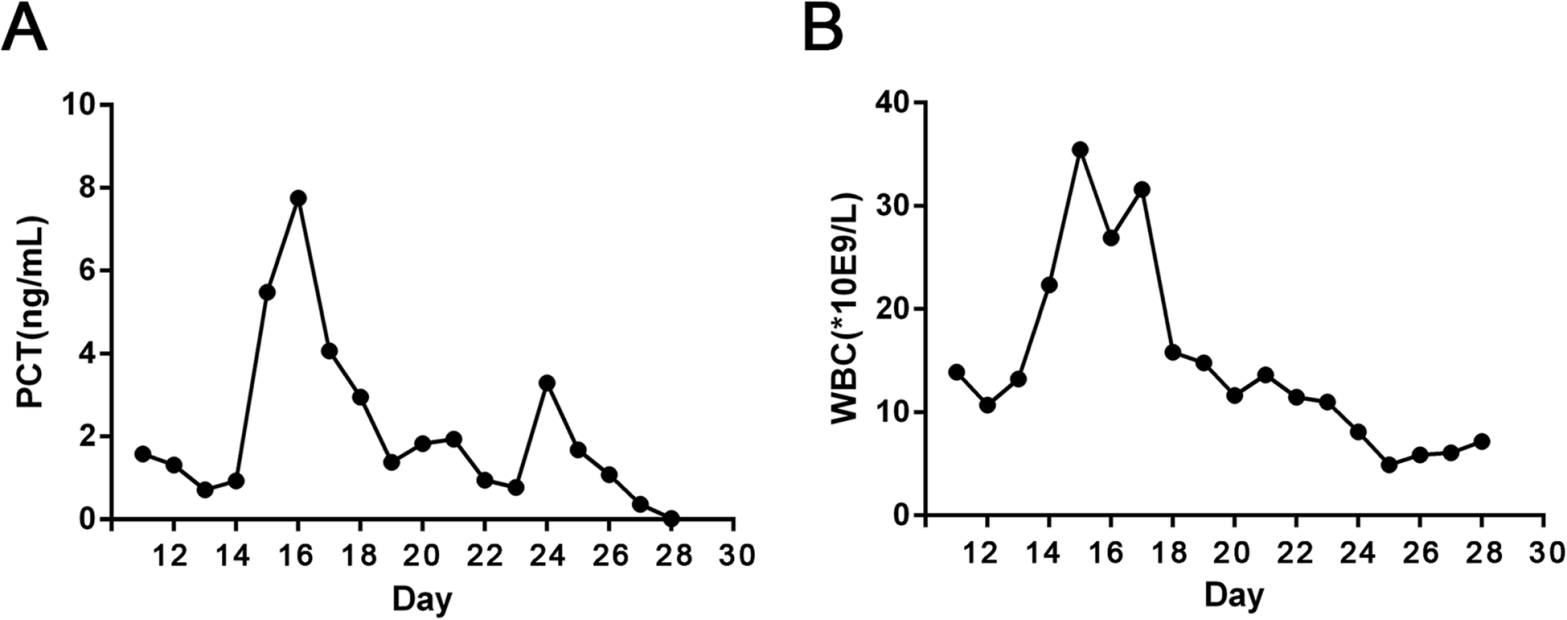
**A**, **B** The changes in procalcitonin and white blood cells from 11 to 28 days post trauma

His pneumonia was severe and septic shock was present. Chest CT showed multiple lung effusion with pleural effusion 8 days post trauma (Fig. 4B), so thoracic close drainage and mechanical ventilation was performed. The maximum temperature was up to 40 °C, and blood pressure was reduced to 80/55 mmHg. Abnormal laboratory findings included PCT at 8 ng/ml, peripheral leukocyte count of 36 × 10^9^/L (Fig. 5A, B). The patient got received anti-sepsis bundles in NICU. Firstly, fluid resuscitation was done (30 ml/kg) within 3 h and mean blood pressure was increased to 65 mmHg by norepinephrine (10 μg kg^−1^ min^−1^). Secondly, sputum culture was done before imipenem was used initially (1 g intravenous (IV), Q8h). Five days later, antibiotics were adjusted to vancomycin (1 g IV, Q12h) because result of sputum culture was MRSA. Thirdly, airway management was performed strictly, including turning over and slapping his back for sputum draining, subglottic aspiration, and to maintain balloon pressure of endotracheal tube up to 25 cm H_2_O to prevent aspiration of oral secretion. The pneumonia and septic shock were improved remarkably 14 days post anti-sepsis bundles (Fig. 4C).

After the pneumonia was cured and withdraw mechanical ventilation successfully, the fracture of right femoral shaft, tibia, and fibula were surgically fixed by orthopedic surgeon. Then hyperbaric oxygen therapy was supplement to the therapeutic regimen and the patient got conscious gradually.

The patient was awake 90 days post trauma. After 2 months of rehabilitation treatment, motor and language skills are fully restored. Then, he was discharged home to continue ambulatory rehabilitation. At the 1 year of follow-up, his modified Rankin Scale (mRS) was 2.

## Discussion

A typical clinical feature of fat embolism syndrome (FES) was a triad of pulmonary, central nervous system (cerebral fat embolism), and cutaneous manifestations several hours post major trauma [1, 4]. Fat emboli can pass through the pulmonary vasculature, resulting in systemic embolization, most commonly in the brain (CFE) [5].

We presented the complicated CFE case with PSH syndrome and septic shock post trauma. He was cured in NICU by intensive care and rehabilitation treatment and got a good outcome (mRS 2) finally.

CFE is highly variable and nonspecific including headache, lethargy, irritability, delirium, stupor, convulsions, or coma [6]. In this case, we spend some time to differentiate CFE with diffuse axion injury (DAI) or seizures post trauma. We conclude the diagnosis of CFE based on the clinical feature and neuroimaging characteristics of CFE. The clinical feature was conscious on admission (GCS 15) and then fall into coma (GCS 6), suggesting the coma was secondary. If he got DAI, coma was immediate and primary. Secondly, he was complicated right closed femoral midshaft femur, tibia, and fibula fracture, which was had risk factor of CFE occurring. Thirdly, characteristics of CFE and DAI were different in terms of MRI. Rutman et al. studied the differences of neuroimaging between CFE and DAI based on MRI analysis according to number, size/shape, and the distribution of microhemorrhages. It is found that CFE had significantly more hemorrhages than DAI, particularly in the frontal, parietal, and occipital lobes; the corpus callosum; and the cerebellum. CFE microhemorrhages were punctate/round, whereas DAI hemorrhages were medium sized and linear. DAI is more likely to demonstrate hemorrhages larger than punctate, which is found in CFE. Diffuse confluent diffusion restriction favors CFE, whereas a few scattered foci favor DAI [7]. Thus, our diagnosis of CFE was mainly based on his clinical feature and the MRI feather. There are no definitive or specific treatments of FES; therefore, management is entirely supportive. Firstly, because FES may quickly progress to respiratory failure, early detection is important so that supportive treatment can be started promptly. In high-risk patients, respiratory status should be monitored closely via continuous pulse oximetry and ABG analysis. Initial or mild FES is treated with supplemental oxygen, titrated to maintain normal PaO2. FES that progresses to respiratory failure requires mechanical ventilation [8, 9]. Secondly, in patients exhibiting neurologic complications, neurologic status should be closely monitored via frequent Glasgow Coma Scale assessments and serial neurologic examinations. Intracranial pressure monitoring may be considered in patients with cerebral edema. Analgesia and sedation should be used judiciously and in conjunction with a sedation/agitation scale to optimize both patient comfort and neurologic exam. Neuromuscular blocking agents should be avoided if possible [10]. Thirdly, intravascular volume should be maintained to limit shock states, because shock may exacerbate lung injury. Albumin has been recommended for volume resuscitation because it binds to fatty acids and, therefore, may decrease the extent of lung. In patients who develop obstructive shock and right ventricular failure, dobutamine, rather than norepinephrine, may be a more effective agent for hemodynamic support due to its increased inotropic properties.

PSH syndrome is similar to seizure post head trauma, sepsis, pulmonary embolism, malignant hyperthermia, overdose of sympathomimetic or anticholinergic agents, or autonomic dysreflexia as seen with acute spinal cord injury. Patient’s medical history, signs, laboratory results (creatine kinase, serial serum lactate, and repeated blood cultures) and cEEG monitoring can facilitate the diagnosis of PSH. Treatment of CFE with PSH is based on supportive and symptomatic therapy. Opioids, β-blockers, α1-agonists, and bromocriptine are some of the options available to manage PSH [3].

## Conclusion

This is a complicated case of CFE with PSH and septic shock post trauma at high altitude. It is key that rapid diagnoses and appropriate drug treatment were available as soon as possible. Early neurological rehabilitation played an important role for good outcome.

## Data Availability

Not applicable.

## Abbreviations

CFE: Cerebral fat embolism
PSH: Paroxysmal sympathetic hyperactivity
FES: Fat embolism syndrome
GCS: Glasgow coma score
SOFA: Sequential organ failure assessment
cEEG: Continuous electroencephalogram
CT: Computed tomography
DAI: Diffuse axion injury
MRI: Magnetic resonance imaging
mRS: Modified Rankin Scale
NICU: Neurosurgical intensive care unit
